# Trends in Hookah Use Among New York City Middle and High School Students, 2008–2014

**DOI:** 10.5888/pcd15.170283

**Published:** 2018-02-08

**Authors:** Kristi Roods, John Jasek, Shannon M. Farley

**Affiliations:** 1 New York City Department of Health and Mental Hygiene, Long Island City, New York

## Abstract

We examined trends in hookah use among New York City middle and high school students. We calculated prevalences, linear trends, and odds ratios of ever and current hookah use, by selected demographic variables, using 2008 through 2014 data from the New York State Youth Tobacco Survey. The prevalence of ever hookah use increased overall from 2008 through 2014 (8.9% to 13.0%, *P* = .01); current use was stable during this period but increased across many demographic characteristics. Our results indicate a need for efforts to educate populations with increasing prevalence of hookah use as well as policies that regulate use to reduce and denormalize hookah smoking.

## Objective

Hookah use has increased among US adolescents and young adults (1–4). Attractiveness of flavored shisha, misperceptions about hazards posed by hookah use and types of shisha being served, and proliferation and limited regulation of hookah establishments contribute to this trend (5–8). Many hookah establishments claim to sell nontobacco shisha; however, research shows it is typically not tobacco-free (8). Even smoke from nontobacco shisha contains many toxic agents found in cigarette smoke (9). We examined trends in hookah use among New York City middle and high school students to better understand hookah use and guide policy.

## Methods

The Youth Tobacco Survey (YTS) is a cross-sectional survey developed by the Centers for Disease Control and Prevention in conjunction with US states. YTS provides data on trends in youth tobacco use, access, and perceptions. New York State has conducted the survey biennially since 2000 and has included questions assessing hookah use since 2008. From 2008 through 2014 the survey was administered to a sample of 4,500 to 6,500 students in randomly selected New York City public and private school classrooms from grades 6 through 12. Participation rates (the school response rate multiplied by the student response rate) ranged from 70% to 83% during these survey years. All data were self-reported and were weighted to adjust for sex, race/ethnicity, and age (10).

Students were asked if they had ever used a hookah or waterpipe to smoke tobacco (ever hookah use) and if they had used a hookah or waterpipe to smoke tobacco at least one day in the past 30 days (current hookah use). Demographic variables assessed were school level (high school or middle school), sex (male or female), and race/ethnicity (Hispanic/Latino, white, black, or Asian). We computed prevalence of hookah use from 2008 through 2014 by demographic characteristics and by cigarette smoking status (never, former, or current). Former cigarette smoking was defined as having tried cigarettes but no use in the past 30 days. Current cigarette smoking was defined as having smoked at least once in the past 30 days. We tested for linear trend across years using *t* tests (significance set at *P* < .05). We computed adjusted odds ratios (AORs) for ever and current hookah use among demographic and cigarette use variables. We used logistic regression models to compute AORs and corresponding 95% confidence intervals (CIs). Analyses were conducted by using SAS version 9.4 (SAS Institute, Inc).

## Results

The prevalence of ever hookah use increased overall from 2008 through 2014 (8.9% to 13.0%, *P* = .01). It also increased among middle school students (2.9% to 8.5%, *P* < .001), female students (8.0% to 16.2%, *P* < .001), Hispanic or Latino students (7.1% to 17.7%, *P* < .001), black students (3.2% to 9.6%, *P* < .001), and former (13.2% to 43.5%, *P* < .001) and never cigarette smokers (2.1% to 6.2%, *P* < .001). In 2014, Hispanic or Latino students had higher odds of ever using hookah than all other racial/ethnic groups. Female students (AOR = 2.6; 95% CI, 1.8–3.9) had higher odds of ever using hookah than male students. Former (AOR = 11.9; 95% CI, 8.8–16.2) and current cigarette smokers (AOR = 22.3; 95% CI, 13.0–38.5) had higher odds of ever use than never cigarette smokers. The odds of ever using hookah among middle school students (AOR = 0.7; 95% CI, 0.5–1.1) were not significantly different from odds of use among high school students (Table 1).

**Table 1:** Prevalence and Adjusted Odds of Ever Hookah Use Among New York City Middle and High School Students, by Selected Demographic Characteristics, New York Youth Tobacco Survey 2008, 2010, 2012, and 2014

Characteristic	2008		2010		2012		2014			*P* Value^c^
	No.^a^	% (95% CI)	No.^a^	% (95% CI)	No.^a^	% (95% CI)	No.^a^	% (95% CI)	AOR^b^ (95% CI)	
**School level**										
**Overall**	57,000	8.9 (5.9–12.0)	61,000	11.1 (8.4–13.8)	82,000	13.8 (10.8–16.7)	72,000	13.0 (10.8–15.2)	—	.01
**School level**										
High school	47,000	13.1 (8.3–17.9)	50,000	15.6 (11.6–19.5)	62,000	18 (14.1–21.9)	51,000	16.1 (13.4–18.9)	1.00 [Ref]	.19
Middle school	8,000	2.9 (1.9–4.0)	10,000	4.4 (3.3–5.6)	19,000	7.8 (6.2–9.3)	20,000	8.5 (6.7–10.3)	0.72 (0.5–1.1)	<.001
**Sex**										
Male	32,000	9.8 (6.2–13.5)	30,000	11.1 (8.0–14.2)	41,000	13.5 (10.3–16.6)	22,000	9.2 (7.0–11.4)	1.00 [Ref]	.95
Female	25,000	8.0 (4.5–11.5)	32,000	11.2 (8.3–14.0)	41,000	14.1 (10.3–17.9)	50,000	16.2 (13.4–18.9)	2.62 (1.8–3.9)	<.001
**Race/ethnicity**										
Hispanic/Latino	16,000	7.1 (4.5–9.7)	24,000	11.9 (8.5–15.3)	40,000	18.4 (14.5–22.3)	37,000	17.7 (13.7–21.8)	1.00 [Ref]	<.001
White	25,000	20.3 (13.1–27.6)	22,000	19.6 (12.5–26.7)	22,000	18.9 (12.4–25.4)	10,000	11.8 (7.4–16.1)	0.62 (0.4–0.9)	.06
Black	6,000	3.2 (1.6–4.7)	6,000	4.0 (2.1–5.9)	9,000	5.9 (3.9–7.9)	15,000	9.6 (7.7–11.5)	0.60 (0.4–0.8)	<.001
Asian	6,000	10.6 (6.4–14.7)	6,000	9.6 (7.0–12.3)	6,000	9.5 (6.5–12.5)	5,000	7.2 (4.6–9.8)	0.39 (0.2–0.7)	.19
**Cigarette smoking status**										
Never	9,000	2.1 (1.5–2.7)	14,000	3.5 (2.5–4.5)	24,000	5.3 (3.9–6.8)	28,000	6.2 (4.7–7.8)	1.00 [Ref]	<.001
Former	15,000	13.2 (10.1–16.3)	23,000	27.7 (21.9–33.6)	23,000	28.5 (23.6–33.4)	24,000	43.5 (37.0–50.0)	11.92 (8.8–16.2)	<.001
Current	24,000	40.3 (36.4–44.2)	17,000	51.7 (42.2–61.1)	21,000	66.9 (58.6–75.1)	10,000	60.6 (47.9–73.2)	22.35 (13.0–38.5)	.05

Abbreviations: — , does not apply; AOR, adjusted odds ratio; CI, confidence interval; Ref, reference.

a Weighted n is rounded to the nearest thousand.

b AORs calculated by using a logistic regression model, adjusting for school level, sex, race/ethnicity, and cigarette use.

c *P* value for linear trend calculated by using *t* test; significance set at *P* < .05.

Current hookah use was flat overall (4.8% to 5.5%, *P* = .33) but increased among middle school (1.6% to 4.5%, *P* < .001), female (3.8% to 6.9%, *P* = .005), Hispanic or Latino (3.6% to 8.3%, *P* < .001), and black students (2.0% to 3.5%, *P* = .04). It decreased among white (10.9% to 5.2%, *P* = .01) and Asian students (5.6% to 2.4%, *P* = .049). The prevalence also increased among former (4.8% to 14.3%, *P* = .002) and never cigarette smokers (0.7% to 2.3%, *P* < .001). In 2014, female students (AOR = 2.5; 95% CI, 1.4–4.5) had higher odds of current hookah use than male students. Former (AOR = 7.1; 95% CI, 3.6–14.0) and current cigarette smokers (AOR = 30.9; 95% CI, 19.0–50.3) had higher odds than never cigarette smokers. The odds were significantly lower among black (AOR = 0.47; 95% CI, 0.3–0.8) and Asian (AOR = 0.4; 95% CI, 0.2–0.8) students than among Hispanic or Latino students; odds of hookah use between white students and Hispanic or Latino students was not significant (AOR = 0.6; 95% CI, 0.3–1.2). We found no significant difference in odds between middle (AOR = 1.2; 95% CI, 0.6–2.5) and high school students (Table 2).

**Table 2:** Prevalence and Adjusted Odds of Current Hookah Use Among New York City Middle and High School Students, by Selected Demographic Characteristics, New York Youth Tobacco Survey 2008, 2010, 2012, and 2014

Characteristic	2008		2010		2012		2014			*P* Value^c^
	No.^a^	% (95% CI)	No.^a^	% (95% CI)	No.^a^	% (95% CI)	No.^a^	% (95% CI)	AOR^b^ (95% CI)	
**Overall**	30,000	4.8 (3.2–6.5)	30,000	5.5 (3.9–7.1)	38,000	6.5 (5.0–8.0)	30,000	5.5 (4.4–6.7)	—	.33
**School level**										
High school	25,000	7.0 (4.6–9.4)	25,000	7.7 (5.4–10.1)	28,000	8.1 (5.9–10.3)	20,000	6.3 (4.5–8.0)	1.00 [Ref]	.70
Middle school	4,000	1.6 (0.9–2.4)	5,000	2.2 (1.3–3.0)	10,000	4.1 (3.0–5.1)	10,000	4.5 (3.3–5.8)	1.23 (0.6–2.5)	<.001
**Sex**										
Male	18,000	5.8 (3.6–8.0)	15,000	5.5 (3.8–7.3)	20,000	6.6 (5.0–8.2)	9,000	3.9 (2.6–5.2)	1.00 [Ref]	.24
Female	12,000	3.8 (2.4–5.3)	15,000	5.5 (3.8–7.1)	18,000	6.4 (4.4–8.3)	21,000	6.9 (5.2–8.5)	2.50 (1.4–4.5)	.005
**Race/ethnicity**										
Hispanic/Latino	8,000	3.6 (2.0–5.2)	13,000	6.6 (3.9–9.2)	20,000	9.3 (7.0–11.6)	17,000	8.3 (6.4–10.1)	1.00 [Ref]	<.001
White	14,000	10.9 (7.3–14.6)	11,000	10.2 (6.3–14.0)	10,000	8.3 (4.6–12.0)	4,000	5.2 (2.5–7.9)	0.60 (0.3–1.2)	.01
Black	3,000	2.0 (1.2–2.9)	2,000	1.1 (0.3–1.8)	3,000	2.1 (0.9–3.4)	5,000	3.5 (2.0–4.9)	0.47 (0.3–0.8)	.04
Asian	3,000	5.6 (2.8–8.3)	3,000	4.4 (2.7–6.0)	3,000	4.0 (1.9–6.1)	1,000	2.4 (0.8–4.0)	0.41 (0.2–0.8)	.049
**Cigarette smoking status**										
Never	3,000	0.7 (0.2–1.1)	6,000	1.4 (0.8–2.0)	9,000	2.0 (1.2–2.8)	10,000	2.3 (1.5–3.1)	1.00 [Ref]	<.001
Former	5,000	4.8 (3.3–6.2)	9,000	10.8 (7.0–14.6)	7,000	9.1 (6.0–12.2)	8,000	14.3 (9.1–19.5)	7.11 (3.6–14.0)	.002
Current	17,000	34.4 (28.4–40.4)	11,000	34.9 (26.0–43.8)	12,000	38.6 (26.7–47.6)	6,000	40.9 (33.1–48.7)	30.9 (19.0–50.3)	.15

Abbreviations: — , does not apply; AOR, adjusted odds ratio; CI, confidence interval; Ref, reference.

a Weighted n is rounded to the nearest thousand.

b AORs calculated by using a logistic regression model adjusting for school level, sex, race/ethnicity, and cigarette use.

c *P* value for linear trend calculated by using *t* test; significance set at *P* < .05.

## Discussion

Our study results show that ever and current hookah use among middle school, female, and Latino and black students increased over time, while current use among white and Asian students decreased. The most disconcerting changes were among female and Hispanic or Latino students, among whom use increased significantly since 2008. These data differ from those of earlier studies of students from 2005 through 2008 (1,2), which showed more prevalent hookah use among white and male students, and studies nationally and statewide from 2010 through 2012 (3,4), which showed closing gaps in the prevalence of hookah use between Latino and white students and between male and female students.

Hookah use is increasing among never and former cigarette smokers. Focus groups among urban young adults demonstrated extensive awareness of alternative tobacco products but disagreement about associated harms (7). Another study showed that hookah use among non–cigarette smokers predicted cigarette smoking initiation, current cigarette smoking, and higher intensity of cigarette smoking at 2-year follow-up, suggesting that hookah use is a precursor to cigarette smoking (11).

Limitations to our study include that YTS is cross-sectional, provides only population snapshots, and collects self-reported data; therefore, results are subject to bias. However, because YTS has collected data on hookah use from both private and public schools since 2008, it is the best available data source and is representative of New York City students.

Our results indicate that hookah use among middle school and high school students in New York City is proliferating, and efforts should be made to educate populations with increasing prevalence. Policies that limit or regulate hookah use, such as increasing taxes to create parity with other tobacco products and extending the city’s smoking ban to regulate nontobacco shisha in public establishments to limit access and denormalize the practice, are suggested. These policies have been implemented across jurisdictions nationally (12). Jurisdictions considering cigarette-specific legislation should also monitor changes in alternative products such as hookah and e-cigarettes.
